# Knocking-Down Cyclin A_2_ by siRNA Suppresses Apoptosis and Switches Differentiation Pathways in K562 Cells upon Administration with Doxorubicin

**DOI:** 10.1371/journal.pone.0006665

**Published:** 2009-08-17

**Authors:** Xiaohui Wang, Yujun Song, Jinsong Ren, Xiaogang Qu

**Affiliations:** Division of Biological Inorganic Chemistry, State Key Laboratory of Rare Earth Resources Utilization, Changchun Institute of Applied Chemistry, Graduate School of the Chinese Academy of Sciences, Chinese Academy of Sciences, Changchun, Jilin, China; Cairo University, Egypt

## Abstract

Cyclin A_2_ is critical for the initiation of DNA replication, transcription and cell cycle regulation. Cumulative evidences indicate that the deregulation of cyclin A_2_ is tightly linked to the chromosomal instability, neoplastic transformation and tumor proliferation. Here we report that treatment of chronic myelogenous leukaemia K562 cells with doxorubicin results in an accumulation of cyclin A_2_ and follows by induction of apoptotic cell death. To investigate the potential preclinical relevance, K562 cells were transiently transfected with the siRNA targeting cyclin A_2_ by functionalized single wall carbon nanotubes. Knocking down the expression of cyclin A_2_ in K562 cells suppressed doxorubicin-induced growth arrest and cell apoptosis. Upon administration with doxorubicin, K562 cells with reduced cyclin A_2_ showed a significant decrease in erythroid differentiation, and a small fraction of cells were differentiated along megakaryocytic and monocyte-macrophage pathways. The results demonstrate the pro-apoptotic role of cyclin A_2_ and suggest that cyclin A_2_ is a key regulator of cell differentiation. To the best of our knowledge, this is the first report that knocking down expression of one gene switches differentiation pathways of human myeloid leukemia K562 cells.

## Introduction

Tumor cells are characterized by deregulation of cell cycle checkpoints, leading to uncontrolled cell division and proliferation under conditions where non-transformed cells cannot enter and pass through the cell cycle. All these may come from over-expression of cyclins and the abnormal activation of cyclin-dependent kinases (CDKs) [1]. Cyclins are a superfamily of proteins whose levels vary in a cyclical fashion during the cell cycle to activate specific CDK required for the proper progression through the cell cycle. Cyclin A_2_, which is essential for initiation and progression of DNA replication as well as for cell cycle progression through G1/S and G2/M transitions [2]–[5], is over-expressed in a variety of human cancers compared with normal cells and tissues [6]–[15]. Deregulated expression of cyclin A_2_ seems to be closely associated with early events in tumor transformation [6], [15]. In addition, its expression level in many types of cancers appears to be of prognostic value such as prediction of aggressiveness, survival or early relapse [9]–[14].

Until recently it has been held that CDK2, presumed master of the known CDK isoforms, is a promising anticancer target for developing small molecule inhibitors. The first-generation CDK inhibitors, flavopiridol and CY-202, are in late-stage clinical trials, and have only modest activity [16]. Recent findings [17], however, suggest that CDK2 may not be a key cell cycle player and question whether selective CDK2 inhibition is a useful cancer therapy strategy. No cell cycle abnormalities are observed in either a CDK2 null mouse or following acute ablation of CDK2 in primary cells, indicating that this gene is not strictly required for cell proliferation [17]. In contrast, deletion of cyclin A_2_ in knockout mice is associated with an embryonic lethal phenotype [18]. Moreover, Fine *et al*. demonstrated that cyclin A_2_ and/or cyclin A_2_- CDK2 complex but not CDK2 is a promising anticancer target with a high therapeutic index [19]. Therefore, inhibitors of CDK2 may not be appropriate for cancer therapy and more efforts are focused on inhibition of cyclin A_2_ and/or cyclin A_2_- CDK2 complex activity. We have shown that reduction of cyclin A_2_ in human chronic myelogenous leukaemia K562 cells using small interfering RNA significantly inhibits cell proliferation [20], further supporting the notion that cyclin A_2_ can serve as a novel therapeutic target.

Apoptosis and differentiation are the predominant two mechanisms by which chemotherapeutic agents kill tumor cells. Low dose of doxorubicin (DOX) induces erythroid differentiation in K562 cells, while high concentration of DOX promotes apoptosis [21]. Although many molecular pathways are involved in the apoptosis-regulatory mechanism, evidences suggest that the cell cycle and apoptosis may be interconnected [22]–[28]. Several studies have shown that increased expression of cyclin A_2_ is found in cells in response to several apoptotic stimuli, but very few studies have dealt with this issue directly [29]–[33]. So it is important to clarify whether the decreased expression of cyclin A_2_ is a cause of cell differentiation or a result of differentiation response.

Carbon nanotubes possess the unique features of being able to enter a living cell without causing its death or without inflicting other damage and can shuttle biological molecules into mammalian cells, indicating their potential application as a vector for the delivery of therapeutic molecules [34]–[36]. Recently we have reported that single wall carbon nanotubes (SWNTs) can induce a sequence-dependent B-A DNA transition [37], selectively induce human telomeric i-motif DNA formation [38], accelerate S1 nuclease cleavage rate [39], cause single-stranded poly(rA) to form a duplex structure and bind to human telomeric i-motif DNA under molecular-crowding conditions [40], [41]. Herein, we explore whether altering the levels of cyclin A_2_ in K562 cells using RNAi delivered by SWNTs can influence cell apoptosis and differentiation induced by chemotherapeutic agent DOX. K562 cells with reduced cyclin A_2_ showed a significant decrease in growth suppression, apoptosis and erythroid differentiation, and were differentiated along megakaryocytic and macrophage-monocytic pathways upon administration with DOX. These findings indicate a positive correlation between cyclin A_2_ and apoptosis induced by DOX and suggest that cyclin A_2_ is a key regulator of cell differentiation, supporting the notion that cyclin A_2_ is an important regulator for cell cycle as well as for cell apoptosis and differentiation. To the best of our knowledge, this is the first report that knocking down expression of one gene can switch K562 cells differentiation pathways.

## Results

### Upregulation of cyclin A_2_ during apoptosis of K562 cells induced by DOX

**Figure 1:**
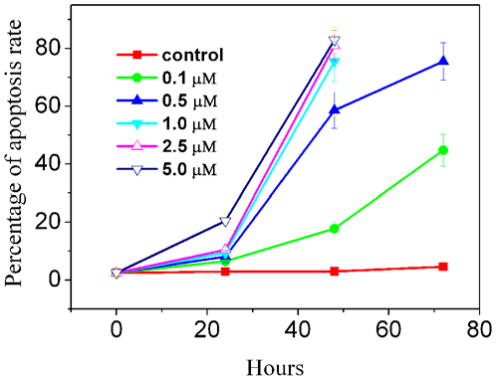
Dose- and time-dependent apoptosis of K562 cells upon administration with DOX. Apoptotic cells were determined by AO/EB staining. Tests were done in triplicate, counting a minimum of 300 total cells from at least three random microscope fields each.

**Figure 2:**
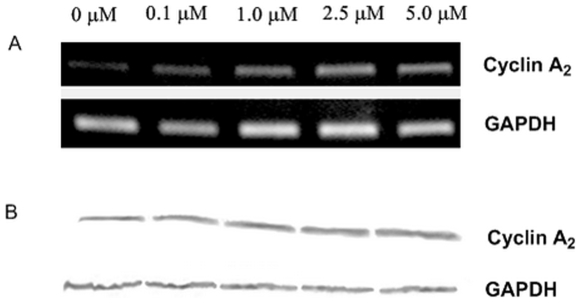
Expression of cyclin A_2_ in K562 cells administered with varying concentrations of DOX. RT-PCR (A) and western blotting (B) were performed 32 hours and 60 hours after drug administration, respectively.

DOX can inhibit growth of a variety of cancer cells [42]. To measure apoptosis rates induced by varying concentrations of DOX in K562 cells, we use acridine orange (AO)/ethidium bromide (EB) staining assay (Fig. 1). The percentage of apoptotic cells increased with time in a dose dependent fashion. RT-PCR and western blotting were used for studying the effect of DOX on the expression of cyclin A_2_ in K562 cells. As shown in Fig. 2, cyclin A_2_ expression levels increased with the increase of DOX, and a positive correlation was observed. These results show that expression of cyclin A_2_ was significantly up-regulated in DOX-treated K562 cells at time points when DOX caused a significant amount of apoptosis, indicating that the levels of cyclin A_2_ are correlated with the ability of DOX to induce apoptosis in K562 cells.

### Suppression of DOX-induced growth inhibition by down-regulation of cyclin A_2_ with siRNA delivered by SWNTs

**Figure 3:**
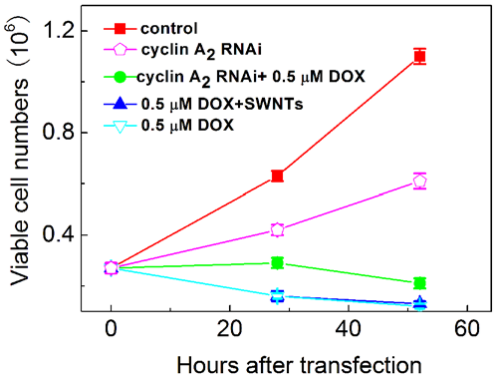
Growth curves of K562 cells in response to cyclin A_2_ siRNA delivered by SWNTs and DOX. The viable cells were counted by trypan blue exclusion at indicated time points. The data shown here represent the average of two independent experiments.

**Figure 4:**
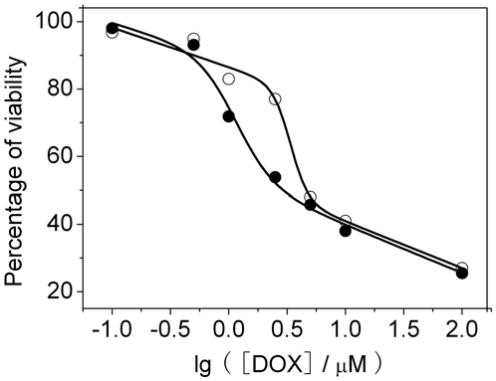
Effect of depletion of cyclin A_2_ on the survival of K562 cells treated with DOX. Cells were transfected with cyclin A_2_ siRNA (○) or not (•) two hours prior to the addition of drug. The viability was assessed by MTT assay as specified in Materials and Methods. The data shown here were means of two separate experiments performed in triplicate.

We have previously demonstrated that functionalized single wall carbon nanotubes (SWNTs) can efficiently deliver siRNA targeting cyclin A_2_ into K562 cells, resulting in specific suppression of cyclin A_2_ expression [20]. Here, we use SWNTs to transfect cyclin A_2_ siRNA into K562 cells and evaluate the effect of cyclin A_2_ on growth inhibition induced by DOX. Two hours after transfection, DOX was added. Cell proliferation was examined by trypan blue exclusion method and methylthiazolyldiphenyl-tetrazolium bromide (MTT) assay. Fig. 3 showed cell growth curves of K562 cells after various treatments. It can be seen that depletion of cyclin A_2_ inhibited cell proliferation while carbon nanotubes vector had no apparent effect on growth inhibition as well as no additive or synergetic effect on cell toxicology of DOX. DOX (0.4 µM) significantly suppressed cell growth, nevertheless, much less growth inhibition was observed in cells incubated with both cyclin A_2_ siRNA and DOX, supported by microscopic results (Fig. S1). In order to further quantitively investigate the effect of down-regulation of cyclin A_2_ on the growth inhibition, MTT assays were carried out 24 h after incubation with DOX. Interaction of DOX with MTT was also checked in a cell free system and the results indicated that DOX showed no inference to MTT assay (Fig. S2). As shown in Fig. 4, IC_50_ of non-transfected cells was about 2.5 µM. For siRNA transfected K562 cells, IC_50_ was about 5.0 µM. The results clearly demonstrate that down-regulation of cellular cyclin A_2_ level suppress DOX-induced growth inhibition.

### Suppression of DOX-induced apoptotic cell death by down-regulation of cyclin A_2_ with siRNA delivered by SWNTs

**Figure 5:**
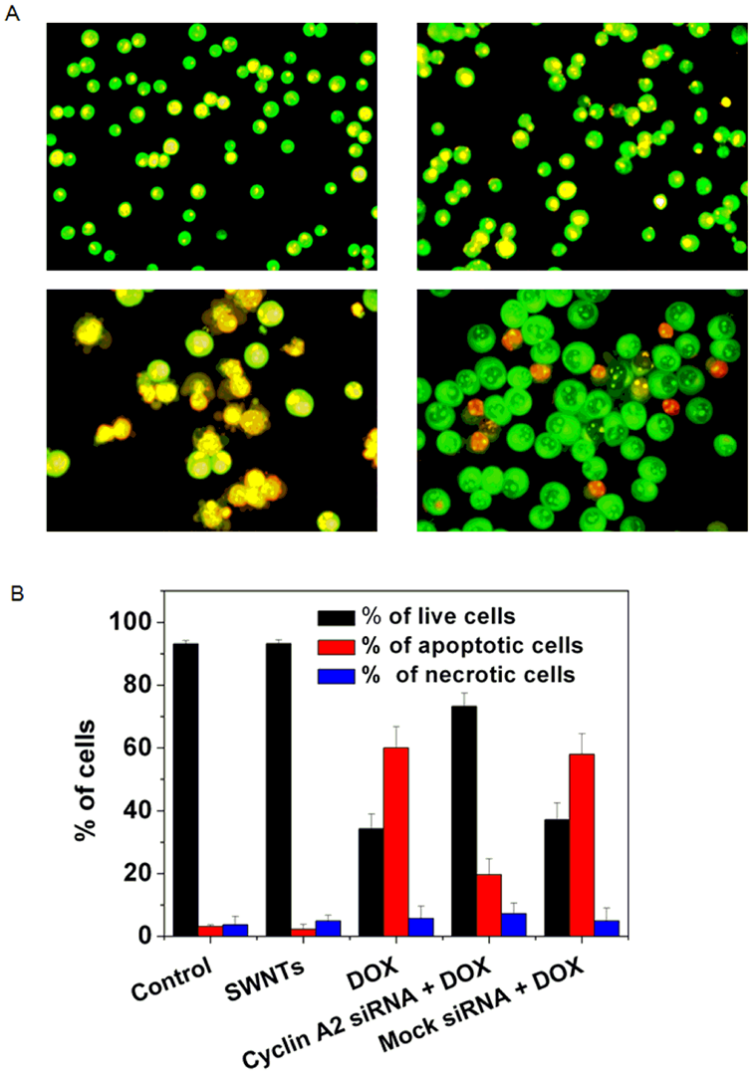
Morphological observation of K562 cells with a fluorescence microscope. Cells were stained with AO/EB dye mixture as described in Materials and Methods section. Shown here were the representative images of three separate experiments. (A): upper left, untreated control cells; upper right, cells treated with transfection vector SWNTs for 32 h; lower left, cells were incubated with 0.4 µM DOX for 32 h, many cells appeared typical apoptotic bleb phenomenon; lower right, two hours after cyclin A_2_ siRNA transfection, cells were then administered with 0.4 µM DOX for additional 32 h. B: quantification of the live, apoptotic, and necrotic cells. Tests were done in triplicate, counting a minimum of 300 total cells each.

To investigate whether cyclin A_2_ participates in cell apoptosis death and determine if reduction of cyclin A_2_ has an effect on apoptosis induced by DOX, siRNA specific for cyclin A_2_ was transfected into K562 cells by SWNTs, and low dose of DOX (0.4 µM) was administered. As shown in Fig. 5, carbon nanotubes vector showed no apparent cell toxicology, while numerous larger cells and typical lobular nuclei were observed in cells incubated with DOX. For cells coadministered with cyclin A_2_ siRNA and DOX, much less apoptotic nuclei were observed, and the size of live cells was much bigger than that of the control cells. Statistics analysis showed that down-regulation of cyclin A_2_ significantly reduced the apoptosis rate from more than 60% to about 20%. Similar results were obtained by annexin V-PI double staining flow cytometric technique (as shown in Figure S3). These clearly indicated lowering cyclin A_2_ level in K562 cells led to suppression of apoptosis, thus a marked decrease in DOX susceptibility, which provide direct evidence that cyclin A_2_ is involved in cellular responses to apoptosis.

### The cytoplasmic subcellular distribution of cyclin A_2_ correlates with apoptosis of K562 cells induced by DOX

**Figure 6:**
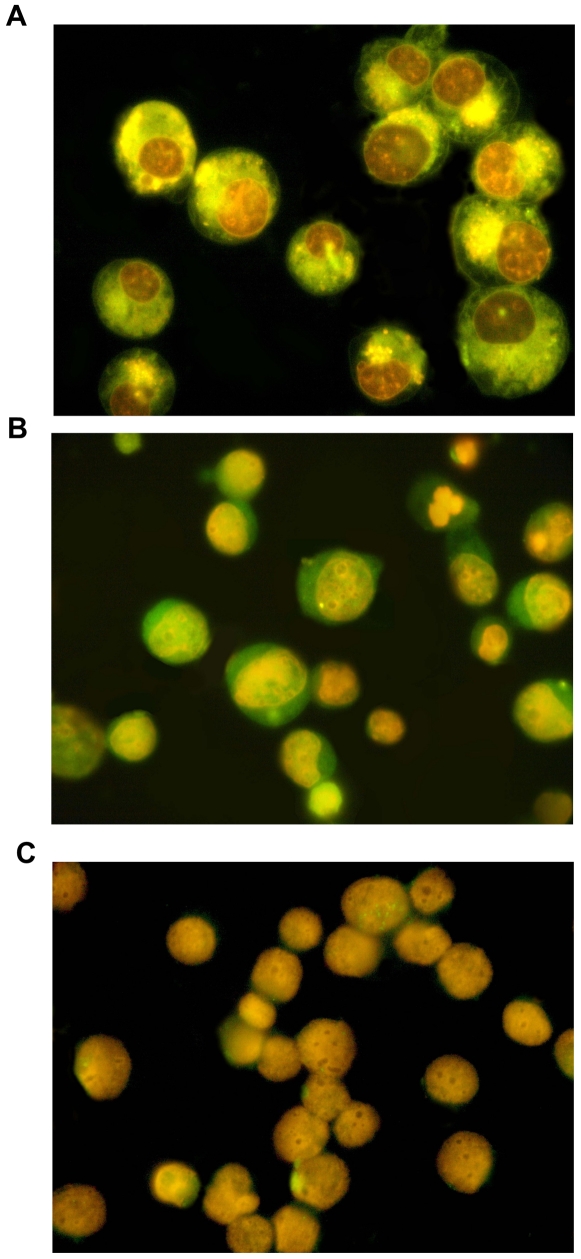
The sub-cellular distribution of cyclin A_2_ in K562 cells correlated with cell apoptosis. Cells were administered with 0.4 µM DOX for 32 h. A significant fraction of cells underwent apoptosis. Immunofluorescence detection of cyclin A_2_ was performed as described in Materials and Methods section. A: representative fluorescence microscopy image of K562 cells after treatment. Orange nuclei were observed, where DOX was mostly located; B: representative immunofluorescence microscopy image of K562 cells. Cyclin A_2_, normally located at nucleus, can be observed in cytoplasm of early and late phases of apoptotic cells. C: representative microscopy image of negative control immunofluorescence in K562 cells. No significant non-specific signal was observed.

Early reports have demonstrated that there is a link between cyclin A_2_ subcellular localization and its cell function, and the level of cyclin A_2_ is correlated to cell apoptosis [22], [23], [26]. For clarifying whether the subcellular distribution of cyclin A_2_ in K562 cells correlates with apoptosis induced by DOX, indirect immunofluorescence detection of cyclin A_2_ was performed. As shown in Fig. 6A, a significant fraction of cells underwent apoptosis and orange nuclei were observed, where DOX was mostly located. The immunofluorescence labeling of cyclin A_2_ showed its presence predominantly in the nucleus of control K562 cells (Fig. S4), whereas in cells administered with DOX, cyclin A_2_ was mainly located at the cytoplasm of early and late phases of apoptotic cells (Fig. 6B). Cyclin A_2_ labeling was not found in the K562 cells incubated with non-immune serum (Fig. 6C). The results indicated that translocation of cyclin A_2_ from the nucleus to cytoplasm was connected with its role in apoptosis.

### Suppression of cyclin A_2_ by siRNA can switch differentiation pathways of K562 cells induced by DOX

**Figure 7:**
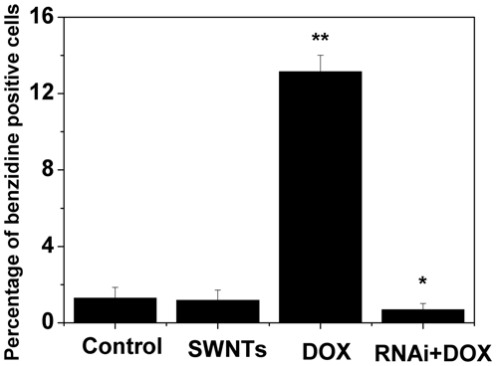
Knocking–down the expression of cyclin A_2_ in K562 cells significantly inhibited erythroid differentiation induced by low concentration DOX. Cells were transfected with cyclin A_2_ siRNA or not two hours prior to the addition of 0.4 µM DOX. Forty hours later, erythroid differentiation was scored by the benzidine staining method to determine the percentage of hemoglobin-positive K562 cells. Tests were done four times, counting a minimum of 300 total cells from at least three random microscope fields each. *p<0.05, **p<0.001 vs. control untreated cells by Student's t-test.

As mentioned above, cells treated with both cyclin A_2_ siRNA and DOX were much bigger than the control. Since enlarged phenotype may suggest cell differentiation, we performed the benzidine staining to assess erythroid differentiation, which was the differentiation pathway of K562 cells upon treatment with anthracycline antibiotics including DOX [42], [43]. Representative microscopy images of the benzidine staining in K562 cells after various treatments were shown in Fig. S5. For untreated cultures and cells administered with SWNTs, the percentages of benzidine positive cells were very low (less than 2%). Forty hours after incubation with DOX, around 14% benzidine positive cells were observed. However, down-regulation of cyclin A_2_ by siRNA in K562 cells substantially suppressed erythroid differentiation upon administration with DOX (less than 1% benzidine positive cells, as shown in Fig. 7).

**Figure 8:**
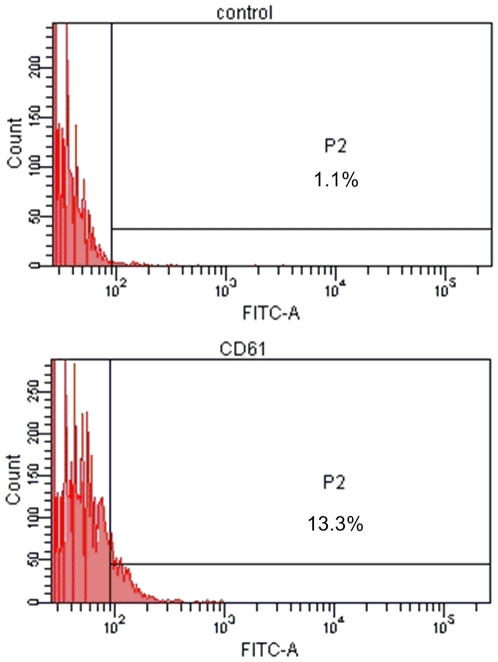
Flow cytometric measurement of megakaryocytic specific surface antigen CD61 (GPIIIa) in K562 cells co-administered with cyclin A_2_ siRNA and DOX. Cells were transfected with siRNA by SWNTs two hours prior to the administration of 0.4 µM DOX. Sixty hours later, expressions of CD61 (GPIIIa) were evaluated by using FITC-conjugated isotype control immunoglobulin and specific anti-CD61 (GPIIIa) FITC-conjugated monoclonal antibody. The marker placed to the right of histogram designates positive events.

**Figure 9:**
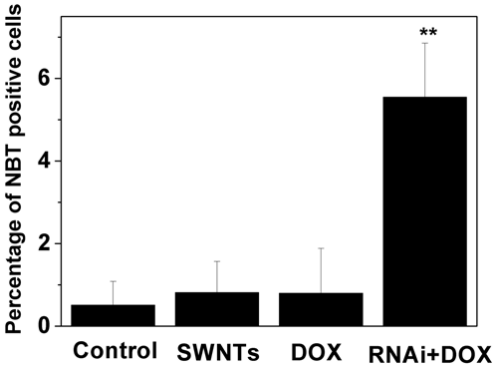
Knocking-down expression of cyclin A_2_ in K562 cells induced monocyte-macrophage differentiation upon administration of DOX. Cells were transfected with siRNA by SWNTs two hours prior to the treatment of 0.4 µM DOX. Ninety six hours later, NBT dye reduction was used to qualitatively monitor monocyte-macrophage differentiation. Tests were done twice, counting a minimum of 300 total cells from at least three random microscope fields each. **p<0.001 vs. control untreated cells by Student's t-test.

To determine whether K562 cells with reduced cyclin A_2_ upon treatment with DOX underwent megakaryocytic pathway and monocyte-macrophage differentiation, which are the other two differentiation pathways of K562 cells, flow cytometric measurement of megakaryocytic specific surface antigen CD61 (GPIIIa) and nitro blue tetrazolium (NBT) reduction assay were carried out, respectively. As shown in Fig. S6, in comparison with the control, cells co-administered with cyclin A_2_ siRNA and DOX showed significant increase in granularity measured by side scatter (side scatter, on *Y*-axis) and cell size measured by forward scatter (forward scatter, on *X*-axis), which was in good agreement with our AO/EB staining observation. Cells treated with DOX or SWNTs showed no detectable expression of CD61 (GPIIIa) (shown in Fig. S7), whereas in K562 cells co-administered with cyclin A_2_ siRNA and DOX, the fraction of CD61 (GPIIIa) positive cells (∼10%) was clearly observed (Fig. 8). For NBT reduction assay, representative microscopy images of K562 cells after various treatments were shown in Fig. S8. In cells treated with DOX or SWNTs, the percentage of NBT positive cells was very low (1%), whereas a small fraction NBT positive cells (∼6%) was observed in K562 cells co-administered with cyclin A_2_ siRNA and DOX (Fig. 9). Although the fractions of cells undergoing megakaryocytic pathway (∼10%) and monocyte-macrophage differentiation (∼6%) were small, the difference is statistically significant compared to the control group. Considering that DOX is an erythroid differentiation-inducing agent for K562 cells [21], [42], [43], it is not surprising that only a small fraction of K562 cells with reduced cyclin A_2_ underwent megakaryocytic pathway and monocyte-macrophage differentiation.

Taken together, these results indicate that knocking down the expression of cyclin A_2_ suppressed DOX-induced erythroid differentiation and a small fraction of K562 cells with reduced cyclin A_2_ were differentiated along megakaryocytic and monocyte-macrophage pathways upon treatment with DOX. These findings suggest that cyclin A_2_ is an important regulator of cell differentiation.

## Discussion

Cyclin A_2_ is particularly interesting among the cyclin family because it can activate two different CDKs and functions in both S phase and mitosis. In S phase, phosphorylated cyclin A_2_-CDK2 complexes are suggested to play an important role in the initiation of DNA replication. In mitosis, cyclin A_2_ may contribute to the control of cyclin B stability. Consistent with its role as a key cell cycle regulator, overexpression of cyclin A_2_ is associated with transformed cells [6]–[15]. However, it is difficult to determine whether elevation of cyclin A_2_ is a contributing factor or a mere consequence of the increased cell proliferation. In haematological malignancies, cyclin A_2_ is associated with proliferation rate of these disorders and can be used for molecular diagnostics as a proliferation marker [13], [14].

Single-walled carbon nanotubes (SWNTs) have been considered as the leading candidate for nanodevice applications ranging from gene therapy and novel drug delivery to membrane separations. We have previously showed that siRNA transfection efficiency of lipofectamine 2000 in K562 cells was low (28%) and some cells underwent apoptosis and necrosis during the process [20]. However, SWNTs could efficiently facilitate the coupling of siRNA to form siRNA∶SWNTs complexes and carry siRNA into K562 cells, significantly knocking down the expression of target gene. No apparent cell toxicology was observed. Hence, in order to directly probe whether cyclin A_2_ participates in cell apoptosis and differentiation, we employed SWNTs as transfection vector to deliver cyclin A_2_ siRNA into K562 cells to specifically knock down the expression of cyclin A_2_ and found that carbon nanotubes vector showed no additive or synergetic effect on cell toxicology of DOX, which was consistent with our previous report [20].

DOX, a prominent member of anthracycline antibiotics, has been extensively used for treatment of solid tumors and leukemia. It exerts its cytotoxic activity against cancer cells mainly by intercalation into DNA, inhibition of topoisomerase II and helicase activity, leading to cell-cycle arrest at the G2/M phase and apoptosis [42]. In clinical applications, doses of DOX are strictly limited by its cardiotoxicity [43]. It should be noted that the dose of DOX administrated here (0.4 µM) is pharmacological relevant compared to the initial or steady-state plasma concentrations observed in patients after standard bolus infusions (5 µM and 25–250 nM, respectively).

It has been reported that there is a link between cyclin A_2_ and apoptosis [22]–[28]. Hoang *et al.* showed that in c-myc overexpressing serum deprived rat 1A fibroblasts undergoing apoptosis, cyclin A_2_ mRNA expression was increased, in contrast to the invariant expression for cyclin B, C, D1 and E [24]. Moreover, serum-deprived rat 1A fibroblast stably transfected with cyclin A_2_ exhibited increased apoptosis following stimulation of cyclin A_2_ expression. Meikrantz *et al.* reported that induction of apoptosis was uniformly associated with activation of cyclin A_2_-dependent kinases but not associated with cyclins E or B, and overexpression of the cyclin A_2_ could circumvent the anti-apoptosis activity of the oncogene BCL-2 in human Hela cells [25], [26]. Furthermore, Hiromura *et al.* indicated that apoptosis was associated with an increase in cytoplasmic cyclin A_2_-CDK2 activity following UV irradiation, under these conditions, nuclear cyclin A_2_-Cdk2 activity decreased significantly [27]. Our results showed knocking down the expression of cyclin A_2_ in K562 cells significantly suppressed the apoptosis induced by DOX and a positive correlation between the levels of cyclin A_2_ and apoptosis was observed. The findings also indicate that the cytoplasmic subcellular distribution of cyclin A_2_ correlates with its pro-apoptotic role. We speculate that cyclin A_2_ associated kinases are involved in DOX-induced apoptotic cell death pathways in K562 cells, although the exact downstream mechanisms are not known.

We have demonstrated that SWNTs could effectively deliver cyclin A_2_ siRNA into K562 cells, significantly suppressing the expression of cyclin A_2_ with specificity and cell proliferation, and cells with reduced cyclin A_2_ showed a decrease in the percentage of cells in S phase [20]. Several studies have indicated that cancer cells with a high S-phase fraction/high proliferative activity are more sensitive to apoptosis induced by chemotherapy [44], [45]. As for DOX, it is active throughout the cell cycle, but the effect is most pronounced for cells in S phase-G2 phase, especially in S phase, where it interferes with the DNA replication and transcription [42]. Therefore, it was not surprising that K562 cells with reduced cyclin A_2_ showed a marked decrease in DOX susceptibility.

Most of chemotherapeutic agents show significant side effects and not all patients benefit from aggressive chemotherapy. Therefore, searching for tumor biological factors which can predict patient prognosis and chemotherapy response would be of most importance. Several studies have indicated that a high level of cyclin A_2_ expression may be a marker of poor prognosis in cancers [10]–[13]. Besides, previous studies have shown that cancer patients with high level of cyclin A_2_ had better chemotherapy response and survival than those with reduced cyclin A_2_ and low expression of cyclin A_2_, indicating that the patients with high expression of cyclin A_2_ are more suitable for chemotherapy [46]–[48]. Our results demonstrate the pro-apoptotic role of cyclin A_2_ in human myeloid leukemia K562 cells, and indicate that cells with low level of cyclin A_2_ were more resistant to chemotherapeutic agent DOX. Poon *et al.* have suggested that a decrease of cyclin A_2_, rather than increase, promotes tumorigenesis, and once the tumor has developed, high levels of cyclin A_2_ simply reflect a high proliferation rate, which can explain this inconsistency [49]. Hence, despite its association with transformed cells, evaluating cyclin A_2_ level in patients will be an important prognostic marker for use of chemotherapy. Patients with high level of cyclin A_2_ may be more responsive to anticancer drugs through the induction of apoptotic cell death. Moreover, it should be cautious to combine doxorubicin chemotherapy with any small molecule drug targeting cyclin A_2_/cyclin A_2_ associated kinases since it can enhance potential drug resistance.

In several systems, it has been reported that down-regulation of cyclin A_2_ and its associated CDK 2 activity are important for successful differentiation [29]–[33]. Ito *et al*. have suggested cyclin A_2_ overexpression is directly related with poor differentiation [31]. Kiyokawa *et al*. have indicated that differentiation of murine erythroleukemia cells induced by hexamethylene is accompanied by a decrease in the level of cyclin A_2_ and CDK2 proteins and the persistent suppression of cyclin A_2_ expression may play a role in HMBA-induced commitment to terminal differentiation [30]. Yoshizumi *et al*. showed down-regulation of cyclin A_2_ gene expression in vivo at both the RNA and protein levels appears to be important in the permanent withdrawal of human and rat cardiomyocytes from the cell cycle during development [32]. Moreover, Rieber *et al*. demonstrated that the interaction of cyclin A_2_ with E2F is the target for tyrosine induction of B16 melanoma terminal differentiation [33]. In this work, we found that knocking down the expression of cyclin A_2_ in K562 cells significantly suppressed DOX-induced erythroid differentiation and a small fraction of cells with reduced cyclin A_2_ were differentiated along megakaryocytic and monocyte-macrophage pathways upon treatment with DOX. To the best of our knowledge, this is the first report that knocking down expression of one gene can switch K562 cells differentiation pathways. The results suggested that cyclin A_2_ is directly involved in the checkpoint of cell differentiation pathways and is a key regulator of this process, although the detail downstream mechanisms are not known. For cancer cells with low level of cyclin A_2_, which are less responsive to chemotherapeutic agents, induction of differentiation might be an alternative strategy. Combination of cyclin A_2_ siRNA and DOX may provide a novel option of such therapeutic strategy.

In conclusion, knocking down the expression of cyclin A_2_ by siRNA delivered by SWNTs suppresses apoptosis and erythroid differentiation, and promotes megakaryocytic and monocyte-macrophage differentiation in human chronic myelogenous leukaemia K562 cells upon administration with DOX. The results demonstrate the pro-apoptotic role of cyclin A_2_ and suggest that cyclin A_2_ is a key regulator of cell differentiation, supporting the notion that cyclin A_2_ is an important regulator for cell cycle as well as for cell apoptosis and differentiation.

## Materials and Methods

### 

#### Ethics statement

The human erythroleukemic cell line K562 [20] was used in this study.

### Chemicals

Doxorubicin (DOX), Acridine Orange (AO), Ethidium Bromide (EB), Methylthiazolyldiphenyl-tetrazolium bromide (MTT), DAPI, Nitro blue tetrazolium (NBT), benzidine and SWNTs (φ = 1.1 nm, purity>90%) were purchased from Sigma-Adrich (St. Louis, MO, USA). Annexin V apoptosis detection kit was obtained from Keygentec (Nanjing, China). The dye mix for the EB/AO staining was 100 µg/mL acridine orange and 100 µg/mL ethidium bromide in phosphate buffered saline (PBS).

### Cell culture

The human erythroleukemic cell line K562 [20] was grown in Iscove's modified Dulbecco's medium (Gibco BRL) supplemented with 10% fetal calf serum in a humidified 37°C incubator with 5% CO_2_. Cells were passed three times per week. DNA fluorochrome staining (DAPI or Hoechst 33258) is used as our routine mycoplasma detection and the cells used for experiments are free of contamination. Exponentially growing cells were used for all experiments described below. Cell viability was determined by trypan blue exclusion in a haemocytometer chamber.

### Transfection

The siRNA oligonucleotides were synthesized by Genepharma Corporation (Shanghai, China). The sequence used for targeting silencing of cyclin A_2_ was 5′-CCAUUGGUC CCUCUUGAUUTT-3′. The nonsilencing control siRNA is an irrelevant siRNA with random nucleotides UUCUCCGAACGUGUCACGUTT. Functionalized single wall nanotubes (f-SWNTs) were prepared according to method described in our previous work [20]. Cyclin A_2_ siRNA: f-SWNTs complexes (w _f-SWNTs_/w _siRNA_ = 40) were added at 25 n mol/L (siRNA concentration) to culture of cells.

### Reverse transcriptase-polymerase chain reaction (RT-PCR)

Total RNA was extracted using Trizol reagent (Invitrogen) according to the manufacturer's instructions. The primers and conditions for cyclin A_2_ were TCCATGTCAGTGCTGAGAGGA (5′), GAAGGTCCATGAGACAAGGC (3′); 94°C for 30 seconds , 60°C for 30 seconds, 72°C for 1 minute for 25 cycles. Three introns are present in this pair of primer so that any contaminating genomic DNA would not be amplified. Primers used for the glyceraldehyde-3-phosphate dehydrogenase (GAPDH) were ACCTGACCTGCCGTCTAGAA (5′), TCCACCACCCTGTTGCTGTA (3′). Two sets of primers were used for each sample, including primers specific for the gene of cyclin A_2_ and primers for GAPDH as an internal control. All PCR products were visualized on 1.5% agarose gel with 0.5 µg mL^−1^ EB.

### Western blotting

For Western blot analysis, cells were lysed in radio-immunoprecipitation assay (RIPA) buffer (150 mmol/L NaCl, 50 mmol/L Tris-HCl, 0.5% sodium deoxycholate, 1% NP-40, 0.1% SDS, pH 7.6) containing protease inhibitors for protein extraction. Protein concentrations were determined using the Bradford assay. 20 µg cell samples were denatured by addition of 2× reducing sample buffer (100 mmol/L Tris, 4% SDS, 25% glycerol, 10% β-mercaptoethanol, 0.01% bromphenol blue, pH 6.8), incubated for 10 minutes at 95°C, and separated on a 12% SDS-PAGE. The proteins were electroblotted to PVDF membrane. After blocking with Tris-buffered saline containing 0.05% Tween 20 (TTBS) and 2% BSA, the membranes were incubated overnight at 4°C with appropriate primary antibody diluted in TTBS. Working dilutions were: 1/500 rabbit polyclonal anti-cyclin A_2_ primary antibody (Lab Vision Corporation, CA, USA); 1/400 anti-GAPDH primary antibody (Santa Cruz, CA, USA). The membranes were washed three times in TTBS for 5 min each and then incubated for 1 h at room temperature with goat anti-rabbit IgG conjugated to horseradish peroxidase (Jackson Immunoresearch, Stratech, UK). After extensive washing in TTBS, the protein–antibody complexes were visualized by CN/DAB substrate according to method described by Yong [50]. Images were photographed using a UVP gel documentation system (Ultraviolet Products, Upland, CA, USA).

### MTT assay

K562 cells were plated in 96-well culture plate at a concentration of 0.5×10^4^ cells/well, and were transfected with cyclin A_2_ siRNA or not by SWNTs. Two hours later, the cells were treated with various concentrations of DOX while the blank control wells were added medium without drug. Cells were then cultured for another 24 hours and 20 µL MTT (5 mg/mL) was added in each well, followed by additional four hour incubation. The supernatants were then discarded carefully and 150 µL dimethylsulphoxide was added and shaken vigorously to dissolve the purple precipitation formation. Optical density (OD) of each well was tested using Bio-Rad model-680 microplate reader with a wavelength of 490 nm.

### Cell fluorescence staining

Cells were collected by centrifugation at 200×*g* for 5 minutes, and then washed twice with PBS. Cell concentration was adjusted as 2×10^6^−5×10^6^ cells/mL. 1 µL EB/AO dye mix was added in 10 µL cell suspension, followed by 10 minutes of incubation in dark. Stained cells suspension were placed on a clean microscope slide and covered with a cover-slip. Cells were viewed and counted using an Olympus BX-51 optical system microscope (Tokyo, Japan) at 400× magnification with a blue filter. Pictures were taken with an Olympus digital camera. We note that the definition is sharper by eye through the microscope than in the photo. Tests were done in triplicate, counting a minimum of 300 total cells from at least three random microscope fields each.

### Indirect immunofluorescence detection

Cells were harvested, washed three times with PBS, fixed with 4% paraformaldehyde in PBS for 30 min at 4°C and permeabilized with 0.5% Triton X-100 in PBS for 5 min at 4°C. After blocking in 10% goat serum, primary cyclin A_2_ antibody (rabbit, polyclonal; Lab Vision Corporation, CA) was diluted to 1∶400. Goat anti-rabbit FITC-conjugated secondary antibody (Jackson Immunoresearch, Stratech, UK) was used at a 1∶200 dilution. If necessary, DAPI was used to visualize cell nuclei. Cells were observed and photographed by fluorescence microscopy with oil immersion objective and appropriate filters.

### Benzidine Staining

Erythroid differentiation was scored by the benzidine staining method for the determination of the percentage of hemoglobin-positive K562 cells induced by low concentration DOX. Briefly, cells were washed twice and then resuspended in 20 µL PBS. 10 µL of benzidine solution (0.2% benzidine, 0.6% H_2_O_2_, 0.5 M acetic acid) was added and incubated for 20 min at room temperature in the dark. Benzidine-positive cells (blue-black staining) were quantitated by light microscopy. At least 300 cells were counted in triplicate for each condition.

### Flow cytometry analysis

Expression of GPIIIa is considered the most selective marker of the megakaryocyte lineage since it is not expressed on cells of other hematopoietic lineages from normal human bone marrow. Hence, staining of cells for surface CD61 (GPIIIa) was used to evaluate megakaryocytic differentiation. It employed a mouse monoclonal antibody fluorescein isothiocyanate (FITC)-conjugated anti-CD61 (eBioscience) or isotype-matched immunoglobulin (IgG1-FITC, eBioscience) at a concentration of 0.6 µg/mL. Cells were harvested, washed, resuspended in PBS containing 10% fetus calf serum and 0.1% NaN_3_ and incubated for 30 min on ice with antibodies in the dark. After washing three times, cells were resuspended in PBS containing 0.5% formaldehyde and 0.1% NaN_3_, and then analyzed on a FACS Arial cytometer (Becton-Dickinson, San Diego, CA, USA). Viable cells were gated using forward and side scatter characteristics. Fluorescence intensity data were acquired using the BD FACSDIVA™ software.

### NBT reduction assay

NBT dye reduction was used to qualitatively monitor monocyte-macrophage differentiation. Briefly, cells were collected, washed with PBS and resuspended in IMDM medium without serum. Cells suspension were mixed with an equal volume of 0.1% NBT dissolved in PBS and incubated at 37°C for 40 min. NBT was reduced to insoluble formazan because of the intracellular oxygen radical release in the cells differentiated to monocytes-macrophages. The percentage of cells containing intracellular reduced blue-black formazan was determined by light microscopy. At least 300 cells per preparation were observed.

### Statistical analysis

Data are expressed as mean±s.d. and analysis of variance was carried out using Student's t test with Origin 7.5 (OriginLab Corporation, Northampton, MA, USA), where *p*<0.05 was considered significant.

## Supporting Information

Figure S1 Knocking-down the expression of cyclin A2 in K562 cells significantly suppressed growth inhibition and apoptosis induced by DOX. Cells were plated in 6-well plate at a density of 0.8×105 cells/mL and transfected with cyclin A2 siRNA (A, B) or not (C, D) by SWNTs two hours prior to the administration of 0.4 µM DOX. 96 hours later, cells were viewed using an inverted microscope with objectives×10 (A, C) and ×40 (B, D). Pictures were taken with an Olympus digital camera. Shown here are the representative images of three independent experiments.
(0.72 MB TIF)

Figure S2 Chemical interaction of DOX with MTT assay. The assay was performed as follows: 200 µL of medium containing DOX at different concentrations was placed in a 96-well plate; 20 µL of MTT solution (5 mg/mL) was added to each well; After incubation for 4 h at 37°C, 150 µL of DMSO was added to each well and absorbance at 490 nm was measured in a Bio-Rad model-680 microplate reader. DOX-free complete medium was used as control and was treated in the same way as the DOX-containing media. Variation (%) = (absorbance of DOX containing medium - absorbance of control)/absorbance of control×100.
(3.08 MB TIF)

Figure S3 Annexin V-PI double staining of cells after various treatments. Upper left, untreated control; upper right, cells treated with transfection vector SWNTs; lower left, cells treated with 0.4 µM DOX for 32 h; lower right, two hours after cyclin A2 siRNA transfection, cells were then administered with 0.4 µM DOX for additional 32 h.
(0.24 MB TIF)

Figure S4 Indirect immunofluorescence detection of cyclin A2 in control K562 cells. DAPI was used to visualize cell nuclei. Cells were viewed using an Olympus BX-51 optical system microscope (Tokyo, Japan) with oil lens and appropriate filters. Representative stained fields are shown: (A), DAPI staining (blue); (B), immunofluorescence detection of cyclin A2 (FITC, green); (C) merged image. As indicated, cyclin A2 was located at the nucleus of K562 cells without DOX treatment.
(0.43 MB TIF)

Figure S5 Representative microscopy images of the benzidine staining of K562 cells after various treatments. Cells were transfected with cyclin A2 siRNA or not two hours prior to the addition of 0.4 µM DOX. Forty hours later, erythroid differentiation was scored by the benzidine staining method as described in Materials and Methods section. Cells were viewed and counted using an Olympus BX-51 optical system microscope (Tokyo, Japan) at 200× magnification. Four independent tests were performed. Pictures were taken with an Olympus digital camera.
(0.65 MB TIF)

Figure S6 Morphological changes of K562 cells after various treatments. Cells were transfected with cyclin A2 siRNA or not two hours prior to the administration of 0.4 µM DOX. Cells were then cultured for another 32 hours. Flow cytometry was used to show changes in size (forward scatter, on X-axis) and granularity (side scatter, on Y-axis). No significant morphological changes of K562 cells were observed upon RNAi mediated by SWNTs compared to the control. Cells administered with DOX underwent G2/M arrest and changes in granularity and cell size were clearly observed. Although knocking-down the expression of cyclin A2 by RNAi significantly inhibited growth suppression and apoptosis induced by DOX, cells co-administered with siRNA targeting for cyclin A2 and DOX showed similar increases in cell size and granularity compared to those treated with DOX alone.
(2.11 MB TIF)

Figure S7 Flow cytometric measurement of megakaryocytic specific surface antigen CD61 (GPIIIa) in K562 cells. Cells were incubated with 0.4 µM DOX (A, B) or SWNTs (C, D) for 60 h. Expressions of CD61 (GPIIIa) were evaluated by using FITC-conjugated isotype control immunoglobulin (A, C) and specific anti-CD61 (GPIIIa) FITC-conjugated monoclonal antibody (B, D). The marker placed to the right of histogram designates positive events. K562 cells administrated with low concentration of DOX or SWNTs did not undergo apparent megakaryocytic differentiation.
(1.96 MB TIF)

Figure S8 Representative microscopy images of the NBT reduction assay of K562 cells after various treatments. Cells were transfected with cyclin A2 siRNA or not two hours prior to the addition of 0.4 µM DOX. Ninety six hours later, NBT dye reduction was used to qualitatively monitor monocyte-macrophage differentiation. Cells were viewed and counted using an Olympus BX-51 optical system microscope (Tokyo, Japan) at 200× magnification. Two independent tests were performed. Pictures were taken with an Olympus digital camera.
(0.34 MB TIF)
