# Supplementary figures and images for: Knocking-Down Cyclin A2 by siRNA Suppresses Apoptosis and Switches Differentiation Pathways in K562 Cells upon Administration with Doxorubicin
Source: PLoS One. 2009 Aug 17;4(8):e6665. doi: 10.1371/journal.pone.0006665 (PMC2721982; doi:10.1371/journal.pone.0006665)

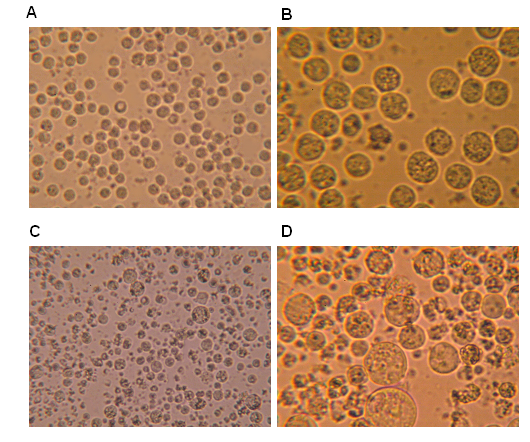

Supplement: Figure S1 — Knocking-down the expression of cyclin A2 in K562 cells significantly suppressed growth inhibition and apoptosis induced by DOX. Cells were plated in 6-well plate at a density of 0.8×10^5^ cells/mL and transfected with cyclin A2 siRNA (A, B) or not (C, D) by SWNTs two hours prior to the administration of 0.4 µM DOX. 96 hours later, cells were viewed using an inverted microscope with objectives×10 (A, C) and ×40 (B, D). Pictures were taken with an Olympus digital camera. Shown here are the representative images of three independent experiments. (0.72 MB TIF) [file pone.0006665.s001.tif]

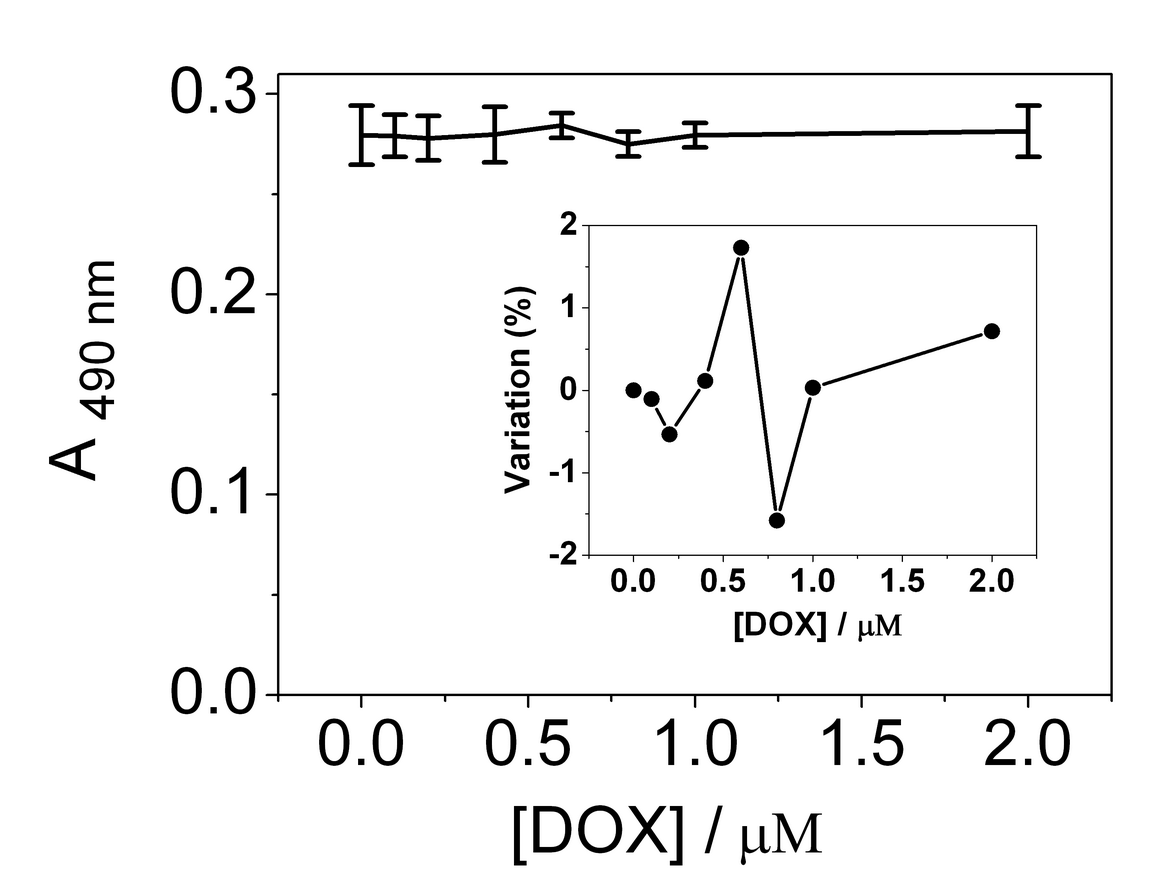

Supplement: Figure S2 — Chemical interaction of DOX with MTT assay. The assay was performed as follows: 200 µL of medium containing DOX at different concentrations was placed in a 96-well plate; 20 µL of MTT solution (5 mg/mL) was added to each well; After incubation for 4 h at 37°C, 150 µL of DMSO was added to each well and absorbance at 490 nm was measured in a Bio-Rad model-680 microplate reader. DOX-free complete medium was used as control and was treated in the same way as the DOX-containing media. Variation (%) = (absorbance of DOX containing medium - absorbance of control)/absorbance of control×100. (3.08 MB TIF) [file pone.0006665.s002.tif]

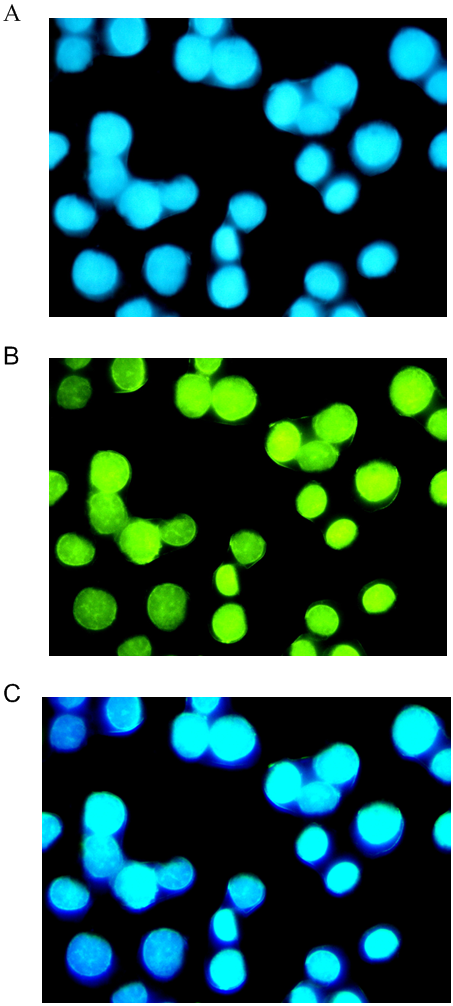

Supplement: Figure S4 — Indirect immunofluorescence detection of cyclin A2 in control K562 cells. DAPI was used to visualize cell nuclei. Cells were viewed using an Olympus BX-51 optical system microscope (Tokyo, Japan) with oil lens and appropriate filters. Representative stained fields are shown: (A), DAPI staining (blue); (B), immunofluorescence detection of cyclin A2 (FITC, green); (C) merged image. As indicated, cyclin A2 was located at the nucleus of K562 cells without DOX treatment. (0.43 MB TIF) [file pone.0006665.s004.tif]

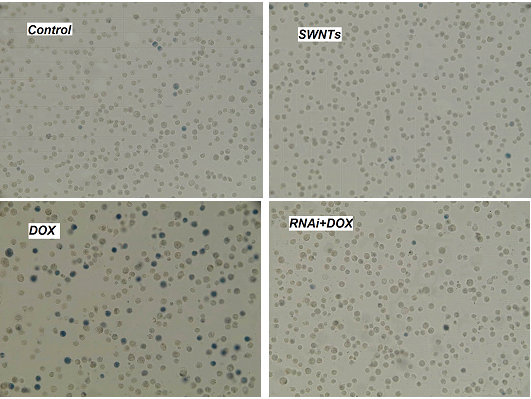

Supplement: Figure S5 — Representative microscopy images of the benzidine staining of K562 cells after various treatments. Cells were transfected with cyclin A2 siRNA or not two hours prior to the addition of 0.4 µM DOX. Forty hours later, erythroid differentiation was scored by the benzidine staining method as described in Materials and Methods section. Cells were viewed and counted using an Olympus BX-51 optical system microscope (Tokyo, Japan) at 200× magnification. Four independent tests were performed. Pictures were taken with an Olympus digital camera. (0.65 MB TIF) [file pone.0006665.s005.tif]

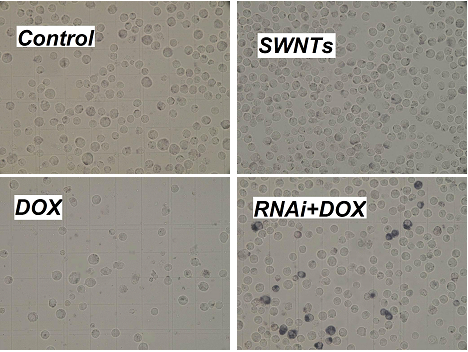

Supplement: Figure S8 — Representative microscopy images of the NBT reduction assay of K562 cells after various treatments. Cells were transfected with cyclin A2 siRNA or not two hours prior to the addition of 0.4 µM DOX. Ninety six hours later, NBT dye reduction was used to qualitatively monitor monocyte-macrophage differentiation. Cells were viewed and counted using an Olympus BX-51 optical system microscope (Tokyo, Japan) at 200× magnification. Two independent tests were performed. Pictures were taken with an Olympus digital camera. (0.34 MB TIF) [file pone.0006665.s008.tif]
